# Prevalence and Associated Factors of Myopia in High-School Students in Beijing

**DOI:** 10.1371/journal.pone.0120764

**Published:** 2015-03-24

**Authors:** Li Juan Wu, Qi Sheng You, Jia Li Duan, Yan Xia Luo, Li Juan Liu, Xia Li, Qi Gao, Hui Ping Zhu, Yan He, Liang Xu, Jost B Jonas, Wei Wang, Xiu Hua Guo

**Affiliations:** 1 School of Public Health, Capital Medical University, Beijing, China; 2 Beijing Key Laboratory of Epidemiology, Capital Medical University, Beijing, China; 3 Beijing Institute of Ophthalmology, Beijing Tongren Eye Center, Beijing Tongren Hospital, Capital Medical University, Beijing Ophthalmology and Visual Science Key Lab, Beijing, China; 4 Beijing Center for Disease Prevention and Control, Beijing, China; 5 Department of Ophthalmology, Medical Faculty Mannheim of the Ruprecht-Karls-University Heidelberg, Seegartenklinik Heidelberg, Germany; 6 School of Medical Science, Edith Cowan University, Perth, Australia; LV Prasad Eye Institute, INDIA

## Abstract

**Purpose:**

To evaluate prevalence and associated factors for myopia in high school students in Beijing.

**Methods:**

Grade 10 and 11 high school students were randomly selected from nine randomly selected districts of Beijing. The students underwent non-cylcoplegic auto-refractometry and an interview.

**Results:**

Out of 4798 eligible students, 4677 (93.4%) students (mean age:16.9±0.7years;range:16–18 years) participated. Mean refractive error of right eyes and left eyes was −2.78±2.29 diopters and −2.59±2.50 diopters, respectively. Prevalence of myopia (defined as ≤ −1.00 diopters in the worse eye) was 80.7% (95% Confidence Interval (CI): 79.6–81.8%). Out of 3773 students with myopia, 1525 (40.4%) wore glasses daily. In multiple logistic regression analysis, a higher prevalence of myopia was associated with female sex (odds ratio (OR) = 1.31;95%CI:1.11–1.55), Han ethnicity (OR = 1.64;95%CI:1.28–2.11), attending key schools (OR = 1.48;95%CI:1.24,1.77), higher family income (OR = 1.37;95%CI:1.09–1.71), longer time spent for near work (OR = 1.43;95%CI:1.06–1.93), shorter near work distance (OR = 1.87;95%CI:1.55–2.26), lower frequency of active rest during studying (OR = 1.40;95%CI:1.16–1.70), and parental myopia (OR = 2.28;95%CI:1.80–2.87). The interaction between distance from near work and time spent for near work was statistically (*P* = 0.03) significant. In multiple logistic regression analysis, higher prevalence of high myopia (≤-6.0 diopters) was associated with studying in key schools (OR = 1.38;95%CI:1.05,1.81), lower frequency of active rest during studying (OR = 1.40;95%CI:1.09,1.79), and a higher number of myopic parents (OR = 2.66;95%CI:2.08,3.40).

**Conclusions:**

A prevalence of about 80% for myopia and a prevalence of about 10% for high myopia in students aged 16 to 18 years and attending classes of grade 10 and 11 in a Chinese metropolitan region is another example of the high prevalence of moderate and high myopia in metropolitan areas of China. With this young myopic generation getting older, myopia as cause for visual impairment and blindness may further increase in importance. Future studies may address whether active rests during studying with looking into the distance are preventive against myopia development or progression.

## Introduction

Myopia has become a global public health problem, in particular in East Asia, where myopic maculopathy and glaucomatous optic neuropathy in association with high myopia have become one of the leading causes of blindness and visual impairment in the elderly population [1,2]. Recent studies on school children in China revealed myopia prevalences ranging between 20% in young children in their early primary school years, and 80% in 17-year-old pupils [3–10]. These studies on school children varied in the study period when the investigations were carried out, in the regions of China where the studies were performed, and whether a mainly urban population or a mainly rural population was included into the investigations. In agreement with the studies on school children, a recent investigation reported a high prevalence of myopia in Chinese university students in Shanghai, with a figure 95% for the myopia prevalence in undergraduate students and a figure of and 97% for postgraduate students [11].

According to the China’s Ministry of Education, there were 24.672 million high school students in China in 2012 [12]. The senior high school education in China follows after a primary school education for 6 years and a junior high school education for 3 years. To enter college, students need to take the National College Entrance Examination (which is also known as “GaoKao”) at the end of the junior high-school years. Since the competition for seats at the university is high, the examination for entrance into the university is difficult and the students intensively prepare for this examination. The high-school students have thus a high load of prolonged near-work during the high-school period during which little time is left for outdoor activities. The China Youth and Children Research Center reported that about 78% of the high school students in China studied more than 8 hours daily at school [13]. This figure was higher than the figure for Korea with 57% of the high-school students with more than 8 hours daily school studying, Japan with 1.1%, and USA with less than 1%. Tang and colleagues reported that only 16% high school students of China have one hour outdoor activity [14]. Previous studies showed that a short duration of outdoors activities is, among other factors such as parental myopia, level of parental education and region of habitation, a major factor associated with the prevalence and incidence of myopia in children [15–24].

We conducted this study to examine the prevalence of myopia and of high myopia in grown-up high school children of grade 10 and 11 in Beijing. Reasons to perform the study were that the group of children in grade 10 and 11 are under particularly high performance and study pressure in the preparation for the final “Gaokao” examination; that recent studies were not specifically focused on that age group; and that the development of myopia is of high importance for public health in East Asia. The latter reason may hold true in particular if one considers that the young myopic generation of today can eventually develop age-dependent myopia-associated complications such as myopic maculopathy and myopic glaucomatous optic neuropathy, when the myopic individuals grow older [25,26].

## Methods

The cross-sectional school based study was performed in Beijing which has an area of 16808 square kilometers and which includes a population of 203477 senior high school students. Sampling for our study was performed in a multi-stage random cluster approach. In a first step, nine districts were randomly selected out of all 18 districts in the rural region and urban region of Beijing. In a second step, one school was randomly selected from each of the nine selected districts. In a third step, all students of grade 10 and 11 of the selected schools with an age of 16 to 18 years were sampled. The sample size was calculated for a prevalence rate of 76% for myopia with a 3% error rate and a 95% confidence interval. The clustering design effect and non-response rate were assumed to be 3% and 10%, respectively. The resulting calculated sample size was 4445. The study was performed one to two months before the final “Gaokao” examination of the high school students. The study was approved by the ethics committee of Capital Medical University, the Beijing Municipal Commission of Education and the Beijing Center for disease Control and Prevention. It followed the tenets of Declaration of Helsinki. Written consent was obtained from the parents of all students.

All students and their parents completed a detailed questionnaire. The quality of the interview was controlled by trained school physicians and disease control officers in each district center. Basic socioeconomic data, such as age, sex, ethnic background, region of habitation (urban/rural), family income per person (<800 RMB, 800–1499 RMB, 1500–2999 RMB, 3000 RMB or more (1 RMB or 1 Yuan equals 0.16 US$)) was collected from the parents, or from the children if in a rare situation the parents did not assist the children in filling out the questionnaire. In a similar manner, information on parental myopia was obtained by directly asking the parents, or in the rare situation of parents not assisting the children in addressing the questionnaire, the students were asked about the glasses of their parents. Data on the use of glasses and the eye condition as diagnosed by an ophthalmologist within the last year was also gathered. Information regarding the type of school was registered. Based on their scores in the senior-high school entrance examination, students in China are assigned to one of two high school types (key schools and non-key schools). Students with higher scores enter the key school and receive a more intensive education.

The questionnaire additionally included questions on near work activities such as the amount of time spent for studying, for watching television and for computer activities; questions on the reading distance (closer than 33 cm (equivalent to one “chi” as a Chinese length unit), or ≥33 cm), the distance when working with a computer (closer than 66 cm (equivalent to two “chis”) or ≥66 cm), and the distance to the television set (closer than 2.5m, or ≥ 2.5m). These questions referred to the period of the two previous months. The total hours of near work per day (defined as the time spent for studying plus time spent for watching television and computer activities) and the distance at which near work was carried out were assessed. If the reading distance, the distance for near work and the distance for computer activities was ≥33 cm and if the distance to the television set was ≥2.5m, the distance was overall classified as adequate. Otherwise, the distance was classified as “close”. We also inquired whether the students had an active rest during their studying periods, and asked about the time spent with sports (indoors or outdoors). The active rest during studying was defined as the students purposely looking far into the distance for ten minutes every 40–50 minutes during their studying periods. It was graded as “occasional” (≤ 5 times every day), as “common” (6–10 times every day), and as “often” (≥11 times every day).

Ophthalmological examinations were performed on the school premises. Non-cycloplegic auto-refractometry (Topcon RM-A7000; Topcon Co., Tokyo, Japan) was carried out by a senior experienced optometrist. The mean of three readings were taken.

The data were analyzed using a commercially available statistical program (SPSS for Windows, version 21.0; IBM-SPSS, Chicago, Illinois, USA). The spherical equivalent of the refractive error was calculated as the spherical refractive error plus half of the minus cylindrical refractive error. Myopia was defined as spherical equivalent of≤-1.00 diopters in the worse eye. High myopia was defined as a refractive error of ≤ -6.0 diopters in the worse eye. The worse eye was defined as the eye with the greater absolute value of the spherical equivalent of the refractive error. The prevalence of myopia was given as mean and 95% confidence intervals (CI). Univariate logistic regression analysis was conducted to examine the relationships between the prevalence of myopia or of high myopia with other parameters such as age, sex, ethnic background, region of habitation, school type and others. A multiple logistic regression analysis using a step-wise backward method was then conducted with the prevalence of myopia as dependent variable and, as independent variables, all factors, which were significantly associated with myopia prevalence in the univariate analysis (probability for stepwise entry 0.05 and removal 0.10). Odds ratios (OR) and 95% (CI) were calculated. All P-values were 2-sided and considered statistically significant when less than 0.05.

## Results

Out of 4798 eligible students, 121 students were excluded because of ocular disorders such as mild conjunctivitis or “red eye” (n = 50), trachoma (mostly inactive) (n = 22), hordeolum (n = 14), keratitis or corneal irritation (n = 11), dry eye syndrome (n = 6), glaucoma (n = 6), scleritis (n = 5), iritis (n = 5), and eye trauma (n = 2). The study finally included 4677 (93.4%) students (2490 (53.7%) girls) with a mean age of 16.9 ± 0.7 years. The majority of the students were Han Chinese (89.3% (Table 1)). Among the study participants, 1374 (29.4%) students were living in the urban region, and 3308 (69.8%) students were living in the rural region; 67.9% of the students were attending key schools.

**Table 1:** Prevalence of Myopia among Senior High-School Students Stratified by Age, Sex, Ethnicity, Region of Habitation, and Type of School.

	Number n (%)	Myopia (Spherical Equivalent of Refractive Error: ≤-1.00D)		High Myopia (Spherical Equivalent of Refractive Error: ≤-6.00D)	
		n (%)	95% Confidence Interval	n (%)	95% Confidence Interval
Age					
16 Years	1449 (31.0)	1190 (82.1)	80.1–84.1	144 (9.9)	8.4–11.4
17 Years	2461 (52.6)	1960 (79.6)	78.0–81.2	248 (10.1)	8.9–11.3
18 Years	767 (16.4)	623 (81.2)	78.4–84.0	70 (9.1)	7.1–11.1
Sex					
Boys	2150 (46.3)	1664 (77.4)	75.6–79.2	203 (9.4)	8.2–10.6
Girls	2490 (53.7)	2078 (83.5)	82.0–85.0	256 (10.3)	9.1–11.5
Ethnicity					
Han	4167 (89.3)	3394 (81.4)	80.2–82.6	426 (10.2)	9.3–11.1
Non-Han	498 (10.7)	370 (74.3)	70.5–78.1	35 (7.0)	4.8–9.2
Region of Habitation					
Urban	1374 (29.4)	1200 (87.3)	85.5–89.1	209 (15.2)	13.3–17.1
Rural	3303 (70.6)	2573 (77.9)	76.5–79.2	253 (7.7)	6.8–8.5
Type of School					
Key School	3177 (67.9)	2666 (83.9)	82.0–85.8	371 (11.7)	10.1–13.3
Non-Key School	1500 (32.1)	1107 (73.8)	71.6–76.0	91 (6.1)	4.9–7.3
Total	4677	3773 (80.7)	79.6–81.8	462 (9.9)	9.0–10.6

Daily studying of more than 8 hours was reported by 3948 (85.3%) students; 3740 (80.1%) students indicated that they had sport of less than one hour daily; 2485 (53.4%) children reported on a commonly or occasionally active rest during daily study.

Mean refractive error for the right eyes and left eyes were-2.78 ± 2.29 diopters and −2.59 ± 2.50 diopters. The overall prevalence of myopia (defined as ≤-1.00 diopters in the worse eye) was 80.7% (95% CI 79.6–81.8%). Out of 3773 students with myopia, 1525 (40.4%) children wore glasses daily, while 1189 (31.5%) students wore glasses occasionally, and 740 (19.6%) students did not have glasses.

In univariate analysis, higher prevalence of myopia was associated with female (OR = 1.47), Han ethnicity (OR = 1.52), urban region of habitation (OR = 1.96), attending key schools (OR = 1.85). higher reported family income (OR = 1.45 for 800–1499RMB; OR = 1.69 for 1500–2999RMB; OR = 2.29 for ≥3000RMB), longer time spent for near work(OR = 1.34 for 9–10.9h; OR = 1.53 for 11–12.9h; OR = 1.63 for ≥13h),shorter distance from which the near work was done (OR = 1.97), lower frequency of active reading during study (OR = 1.51 for common frequency; OR = 1.55 for occasional frequency) and parental myopia (OR = 2.73 for father or mother myopic; OR = 5.93 for both myopic). The prevalence of myopia was not associated with age and sport time (Table 2).

**Table 2:** Associations (Univariate Analysis) between the Prevalence of Myopia (Defined as a Spherical Equivalent of Refractive Error of ≤-1.0), High Myopia (Defined as a Spherical Equivalent of Refractive Error of ≤-6.0 Diopters) and Associated Factors in Senior High-School Students.

	Myopia (Spherical Equivalent of Refractive Error: ≤-1.00D			High Myopia (Spherical Equivalent of Refractive Error: ≤-6.00D		
	Odds Ratio	95% Conf. Interval	*P*-Value	Odds Ratio	95% Conf. Interval	*P*-Value
Age (Years)	0.95	0.85–1.06	0.355	0.97	0.84–1.11	0.63
Sex (Boys Versus Girls)	1.47	1.27–1.71	<0.001	1.10	0.91–1.33	0.34
Ethnicity (Non-Han Versus Han)	1.52	1.22–1.88	<0.001	1.51	1.05–2.16	0.025
Region of Habitation (Rural Versus Urban)	1.96	1.64–2.34	<0.001	2.16	1.78–2.63	<0.001
Type of School (Non-key versus Key School)	1.85	1.60–2.15	<0.001	2.05	1.61–2.60	<0.001
Family Income per Month						
<800	1			1		
800–1499	1.45	1.17–1.80	0.001	1.35	0.94–1.93	0.11
1500–2999	1.69	1.37–2.07	<0.001	2.04	1.48–2.82	<0.001
≥3000RMB	2.29	1.85–2.84	<0.001	2.43	1.78–3.31	<0.001
TSNW						
<9	1			1		
9–10.9	1.34	1.03–1.74	0.028	1.28	0.85–1.94	0.24
11–12.9	1.53	1.17–1.99	0.002	1.35	0.89–2.04	0.16
≥13h	1.63	1.24–2.15	<0.001	1.53	1.005–2.33	0.047
DNW (Adequate versus Close)	1.97	1.66–2.34	<0.001	1.11	0.86–1.43	0.42
Active Rest during Study						
Often	1			1		
Common	1.51	1.27–1.78	<0.001	1.46	1.16–1.82	0.001
Occasional	1.55	1.28–1.89	<0.001	1.53	1.19–1.95	0.001
Sport Time						
<0.5	1			1		
0.5–0.99	1.05	0.90–1.24	0.527	1.19	0.95–1.49	0.13
≥1h	0.96	0.79–1.18	0.713	1.02	0.77–1.36	0.90
Parental Myopia						
Both Not Myopic	1			1		
Father Myopic or Mother Myopic	2.73	2.22–3.36	<0.001	3.01	2.41–3.76	<0.001
Both Myopic	5.93	3.71–9.48	<0.001	6.68	5.09–8.76	<0.001

Abbreviation: TSNW, Time Spent for Near Work; DNW, Distance from Near Work

Multiple logistic regression analysis using the step-wise backward method with the prevalence of myopia as the dependent variable and factors, which were significantly associated with myopia prevalence in the univariate analysis, as independent variables, revealed that a higher prevalence of myopia remained to be statistically associated with female sex (OR = 1.31), Han ethnicity (OR = 1.64), attending key schools (OR = 1.48), higher family income (OR = 1.31 for 800–1499 RMB; OR = 1.37 for 1500–2999RMB; OR = 1.58 for ≥3000RMB), longer time spent for near work (OR = 1.43 for 11–12.9h; OR = 1.39 for ≥13h), shorter distance of near work (OR = 1.87), lower frequency of active rest during study (OR = 1.40 for common frequency; OR = 1.30 for occasional frequency), and parental myopia (OR = 2.28 for father or mother myopic; OR = 4.02 for both parents myopic). In that model, prevalence of myopia was no longer significantly associated with the region of habitation (Table 3; model 1).

**Table 3:** Multiple Logistic Regression Analysis of Associations between the Prevalence of Myopia (Defined as a Spherical Equivalent of Refractive Error of ≤-1.0 and Associated Factors in Senior High-School Students.

	Model 1*			Model 2**		
	Odds Ratio	95% Conf. Interval	*P*-Value	Odds Ratio	95% Conf. Interval	*P*-Value
Sex (Boys Versus Girls)	1.31	1.11–1.55	0.002	1.31	1.11–1.55	0.002
Ethnicity (Non-Han Versus Han)	1.64	1.28–2.11	<0.001	1.64	1.27–2.10	<0.001
Type of School (Non-key versus Key School)	1.48	1.24–1.77	<0.001	1.48	1.24–1.77	<0.001
Family Income per Month						
<800	1			1		
800–1499	1.31	1.04–1.64	0.022	1.30	1.04–1.64	0.02
1500–2999	1.37	1.09–1.71	0.007	1.37	1.09–1.71	0.007
≥3000RMB	1.58	1.24–2.02	<0.001	1.58	1.23–2.01	<0.001
TSNW						
<9	1			-	-	-
9–10.9	1.28	0.95–1.71	0.104			
11–12.9	1.43	1.06–1.93	0.018			
≥13h	1.39	1.02–1.90	0.040			
DNW (Adequate versus Close)	1.87	1.55–2.26	<0.001	1.61	1.28–2.03	<0.001
Active Rest during Study						
Often	1			1		
Common	1.40	1.16–1.70	0.001	1.40	1.16–1.70	0.001
Occasional	1.30	1.04–1.62	0.023	1.30	1.04–1.62	0.02
Parental Myopia						
Both Not Myopic	1			1		
Father Myopic or Mother Myopic	2.28	1.80–2.87	<0.001	2.28	1.80–2.88	<0.001
Both Myopic	4.02	2.42–6.67	<0.001	4.02	2.42–6.66	<0.001
Interaction***						
DNW by TSNW 1 (9–10.9h)				1.13	0.96–1.33	0.14
DNW by TSNW 2 (11–12.9h)				1.21	1.03–1.43	0.02
DNW by TSNW 3 (≥13h)				1.21	1.02–1.43	0.03

Abbreviation: TSNW, Time Spent for Near Work; DNW, Distance from Near Work

*Model 1: Multiple logistic regression analysis using the step-wise backward method of testing associations between myopia and other parameters

**Model 2: Multiple logistic regression analysis using the step-wise backward method of testing association between myopia, other parameters, and the interaction between sex and type of school, interaction between sex and family income per month, and the interaction between DNW and TSNW.

***The dummy variable of TSNW1 = 1, if the observation was for 9–10.9h; the dummy variable of TSNW2 = 1, if the observation was for 11–12.9h; the dummy variable of TSNW3 = 1, if the observation was for 1≥13h.

The interaction between sex and type of school, sex and family income per month, distance from near work and time spent for near work were also explored. The interaction between distance from near work and the dummy variable of time spent for near work 2 (11–12.9h versus <9h) (OR = 1.21), distance from near work and the dummy variable of time spent near work 3 (≥13h versus <9h) (OR = 1.21) were found to be statistically significant. In that model, the combined effect of distance from near work and the time spent for near work were greater than their sum. The prevalence of myopia was also associated with female sex (OR = 1.31), Han ethnicity (OR = 1.64), attending key schools (OR = 1.48), higher family income (OR = 1.30 for 800–1499 RMB; OR = 1.37 for 1500–2999RMB; OR = 1.58 for ≥3000RMB), shorter distance of near work (OR = 1.61), lower frequency of active rest during study (OR = 1.40 for common frequency; OR = 1.30 for occasional frequency), and parental myopia (OR = 2.28 for father or mother myopic; OR = 4.02 for both parents myopic). In that model, prevalence of myopia was no longer significantly associated with time spent for near work (*P*>0.10) (Table 3; model 2).

Analysis of collinearity showed a variance inflation factor of less than 1.5. In the assessment of collinearity, we additionally analyzed a correlation matrix including the independent variables which were significantly with the prevalence of myopia in the multiple logistic regression model. The highest correlation coefficient was 0.31. We finally applied the Hosmer and Lemeshow goodness of fit’ test for the regression model 2 and arrived at a Chi-square of 4.69 with a *P*-value of 0.79, indicating that there was an overall fit of the model.

Mean prevalence of high myopia was 9.9% (95%CI: 9.0–10.6%). In univariate logistic regression analysis, prevalence of high myopia was significantly associated with Han ethnicity (OR = 1.51), urban region of habitation (OR = 2.16), studying in key schools (OR = 2.05), higher family income (OR = 2.04 for 1500–2999 RMB; OR = 2.43 for ≥3000RMB), lower frequency of active rest during studying (OR = 1.46 for common frequency; OR = 1.53 for occasional frequency), longer time spent for near work (OR = 1.53 for ≥13h), and parental myopia (OR = 3.01 for father myopic or mother myopic; OR = 6.68 for both parents myopic) (Table 2). In the multiple logistic regression analysis, higher prevalence of high myopia was associated with studying in key schools (OR = 1.38), lower frequency of active rest during studying (OR = 1.40 for common frequency; OR = 1.32 for occasional frequency), and a higher number of myopic parents (OR = 2.66 for father myopic or mother myopic; OR = 5.65 for both parents myopic) (Table 4). The prevalence of high myopia was no longer significantly associated with ethnicity, family income, urban region of habitation, longer time spent for near work (Table 4).

**Table 4:** Multiple Logistic Regression Model of Factors Associated with the Prevalence of High Myopia (Defined as a Spherical Equivalent of Refractive Error of ≤-6.0 in the Worse Eye) in Senior High-School Students.

	Myopia (Spherical Equivalent of Refractive Error: ≤-1.00D		
	Odds Ratio	95% Conf. Interval	*P*-Value
Region of Habitation (Rural Versus Urban)	1.27	1.00–1.61	0.051
Type of School (Non-key versus Key School)	1.38	1.05–1.81	0.020
Active Rest during Study			
Often	1		
Common	1.40	1.09–1.79	0.008
Occasional	1.32	1.01–1.74	0.043
Parental Myopia			
Both Not Myopic	1		
Father Myopic or Mother Myopic	2.66	2.08–3.40	<0.001
Both Myopic	5.65	4.15–7.70	<0.001

## Discussion

The prevalence of myopia as found in our study population (80.7%; 95% CI: 79.6–81.8%) was comparable with figures reported from Taiwan (myopia prevalence in high schools: 84%) and from the Shanghai Province (myopia prevalence in high schools: 80%) [27,28]. Myopia prevalence in our study population was considerably higher than in a study population from Singapore (myopia prevalence in grade 9 and 10 students: 73.9%) [29], and it was considerably higher than in two Iranian studies with a myopia prevalence of 33.0% and 29.3%, respectively, in school children of grade 10 to 12 [30,31]. In the latter two studies as in our investigation, refractometry had been carried out under non-cycloplegic conditions. The prevalence of myopia in the rural region of our study (77.7%) was higher than that found in children of the same age group in the population-based Yangxi Eye Study performed in the rural region of the Chinese Guangzhou Province (myopia prevalence in 17 years old: 53.9%) (Table 5).[7] Compared with other age groups from the same region, the myopia prevalence in our study population was markedly higher than that found in junior high school students with an age of 15 years (myopia prevalence: 65.1% [10,21].

**Table 5:** Reported Prevalence of Myopia in Previous Studies on High School Children.

Author (Year)	Country	Study design / Sample size for high school(N)	Age (Years)	Cycloplegic Refraction	Myopia Definition	Prevalence
Fotouhi (2006)[30]	Iran	School based / N = 994(grade 10 to 12)	14–18	Non-cycloplegic refraction	≤-0.5D	33.0%
Quek (2004)[29]	Singapore	School based / N = 983(grade 9 to 10)	14–19	Non-cycloplegic refraction	≤-0.5D	73.9%
Lin (2004)[27]	Taiwan	School based / N = not mentioned (senior high school)	16–18	Cycloplegic refraction	≤-0.25D	84.0%
1. Qian (2009)[28]	China (Shanghai, Xinjiang)	Case-control study/ N = 927(senior high school)	15–18	Cycloplegic refraction	≤-0.5D	65.8% Shanghai 80%

In our study, the students with Non-Han ethnicity had a significantly lower prevalence of myopia and high myopia than students with Han ethnicity (Tables 1–3). Qian et al. reported similar results [28]. In a similar manner, a lower prevalence of myopia in students from China’s minority groups as compared to Han Chinese students was found in the investigation by Sun and colleagues who examined 5060 university students in Shanghai and where the ethnic minority groups (such as Uygurs, Buyis, Kazaks, and others) were less myopic than Han students [11]. The ethnic differences in the prevalence of myopia in our study remained significant after adjusting for other parameters such as rural or urban region of habitation, school type, family income, near-work activities, and parental myopia. It has remained unclear, whether these ethnic differences in the prevalence of myopia and high myopia are due to genetic differences or due to differences in lifestyle, with, e.g., Uygurian students having potentially spent more time outdoors in their earlier childhood. Previous studies have shown that the time spent outdoors is protective against the development of myopia [15,17,18,20,21].

In our study, near work was a factor associated with a higher prevalence of myopia. A higher prevalence of myopia was positively associated with a shorter distance from which near work was done. Our study findings are in agreement with the findings from the Sydney Myopia Study, which found children who performed near work at a distance of less than 30 cm were 2.5 times more likely to have myopia than those who worked at a longer distance [32]. Similar findings were reported from military conscripts in Taiwan [23]. Due to the cross-sectional character of our study and the previous studies, however, it remained inconclusive whether a short reading distance was the cause or the consequence of myopia.

In the multivariate in our study (model 1), there was a statistically significant tendency for an association between higher myopia prevalence and longer time spent for near work. Similar findings were reported from Australia, Taiwan, USA, Singapore and China [16,23,33,34]. We also found an interaction between distance from near work and time spent for near work (model 2). Again, it could not be determined whether a long duration of near work including studying was causing myopia, or whether myopic students had the tendency to study more.

Interestingly, we found that a higher prevalence of myopia and high myopia was associated with a lower frequency of active rest during studying. It confirmed a study on Australian school children, in which the usual duration of continuous reading was associated with myopia [32]. The results of our cross-sectional study were also in agreement with the findings of a recent longitudinal investigation in which school children who spent their class recess outdoors as compared to school children who spent it indoors had a significantly lower incidence of myopia (8.4% vs 17.7%) after one year of follow-up [24]. It may suggest that an active rest during studying with looking far into the distance for 10 minutes every 40–50 minutes may be a possibility to reduce the development or progression of myopia for high school students.

We failed to find an association between prevalence of myopia and time spent with sports. It would be in agreement with the findings form Guggenheim and colleagues in whose study the time spent outdoors was predictive of incident myopia independently of the physical activity level [20]. Since we did not assess whether the sport was performed outdoors or indoors, we could not assess whether staying outdoors or sportive outdoors activities were associated with a lower prevalence of myopia as previous studies have shown. The association between parental myopia and myopia of the students as found in our study agrees with numerous investigations [19,32,35,36].

Potential limitations of our study should be mentioned. First, refractometry was performed without cycloplegia to achieve a participation rate of 97.6% in the study. It is in contrast to studies such as the Refractive Error Study in Children [37]. Non-cycloplegic refractometry may overestimate the prevalence of myopia in teenagers with active accommodation [38]. Since, however, we mainly defined myopia as a refractive error of ≤1.0 diopters, since our study was not focused on the prevalence of hyperopia (in the case of which active accommodation may have markedly affected the non-cycloplegic refractometric measurements) and since it may not be very likely that myopic individuals had a large accommodative excess, the weakness in our study design not to use cycloplegia may not have markedly influenced the results of our study. Second, we did not assess the outdoor activity time, which was reported as a protective factor for myopia development. However, since the high school students had a prolonged indoor study time, little time was left for outdoor activity. The inter-individual outdoor activity time difference might be small, hence has a trivial impact in our result. Third, the parameters of reading distance and distance of watching television were self-reported and were measured, and were inaccurate to a certain degree. We used however, the Chinese length unit of “Chi” (1 Chi = 33cm) which has been in common use in the Chinese culture for more than 3000 years and which is known to all Chinese. Fourth, in a similar manner, the data on active rests during studying were subjective. The questions were asked however in a standardized and detailed manner, so that the variability in the subjective responses may have been reduced. Fifth, we did not measure visual acuity of the students, since school doctors regularly examined the uncorrected visual acuity once per year and sent the students to the optician in the case of a reduced visual acuity. Sixth, the questions on activities such as reading, studying and reading distance referred to the period of the two previous months. Since myopia started to developed considerably earlier, it remained unclear how representative the activities of high school students were for their activities when the students were less than 10 years old. Seventh, due to the cross-sectional study design, the present investigation does not allow to make assumptions about causal relationships. Information on time spent for near work, distance for near work, active rest during study period, and time spent for sports could be influenced by myopia and in a reverse manner, could be a causal effect of myopia rather than cause for myopia. The cause effect relationship is difficult to determine from such a cross-sectional study and may be examined in future longitudinal studies.

In conclusion, the prevalence of myopia in our study population of grade 10 and grade 11 students was 80.7% and that for high myopia was 9.9%. Higher prevalence of myopia was significantly associated with female sex, Han ethnicity, attending key schools, higher family income, shorter near work distance, lower frequency of active rest during study, and parental myopia. Future studies may address whether active rests during studying with looking far into the distance for 10 minutes every 40–50 minutes may be a possibility to reduce the development or progression of myopia for high school students.
